# Employers’ attitude, intention, skills and barriers in relation to employment of vulnerable workers

**DOI:** 10.3233/WOR-210898

**Published:** 2022-08-11

**Authors:** G. Hulsegge, W. Otten, H.A. van de Ven, A.M. Hazelzet, R.W.B. Blonk

**Affiliations:** a Sustainable Productivity and Employability, Netherlands Organization for Applied Scientific Research (TNO), Leiden, The Netherlands; b Faculty of Social and Behavioral Sciences, Tilburg University, Tilburg, The Netherlands; c Optentia, North West University, Vanderbijlpark, South Africa

**Keywords:** Disability, employers, hiring intention, retention, occupational rehabilitation

## Abstract

**BACKGROUND:**

Little is known why some organizations employ vulnerable workers and others do not.

**OBJECTIVE:**

To explore the relationships between the attitude, intention, skills and barriers of employers and employment of vulnerable workers.

**METHODS:**

We included 5,601 inclusive organizations (≥1% of employees had a disability, was long-term unemployed or a school dropout) and 6,236 non-inclusive organizations of the Netherlands Employers Work Survey 2014–2019. We operationalized employer factors based on the Integrative Model of Behavioral Prediction as attitude (negative impact), intention (mission statement regarding social inclusion), skills (human resources policies and practices), and barriers (economic conditions and type of work). We used multivariate-adjusted logistic regression models.

**RESULTS:**

Compared to non-inclusive organizations, inclusive organizations had a more negative attitude (OR:0.81) and a stronger intention to employ vulnerable workers (OR:6.09). Regarding skills, inclusive organizations had more inclusive human resources practices (OR:4.83) and initiated more supporting human resources actions (OR:4.45). Also, they adapted more work conditions towards the needs of employees (OR:1.52), negotiated about work times and absenteeism (OR:1.49), and had general human resources practices on, for example, employability (OR:1.78). Inclusive organizations had less barriers reflected by better financial results (OR:1.32), more employment opportunities (OR:1.33) and more appropriate work tasks (OR:1.40).

**CONCLUSIONS:**

Overall, inclusive organizations reported more positive results on the employer factors of the Integrative Model of Behavioral Prediction, except for a more negative attitude. The more negative attitude might reflect a more realistic view on the efforts to employ vulnerable groups, and suggests that other unmeasured emotions and beliefs are more positive.

## 1 Introduction

Employment is an important way to participate in society. Meaningful work meets the basic psychological needs of autonomy, competence and relatedness [1]. Work is, therefore, important to the health and well-being of people in general and in particular of vulnerable workers, which include people with a disability, the long-term unemployed and school dropouts [2, 3]. Aside from the benefits for the individual, organizations also benefit from employing vulnerable workers. It increases profitability (e.g., profits, turnover and retention, and company image) and competitive advantage (e.g., diverse customers, customer loyalty and satisfaction, and innovation) [4]. Despite the benefits of employing vulnerable workers, employment rates of these groups remain remarkably low when compared to the general population [5–8].

Understanding how vulnerable workers can be part of the labor market has been the subject of research for many years. Most studies focused on supply-side factors making people job-ready, such as examining the effectiveness of reintegration, and developing interventions to support people to find work [9]. Currently, there is increasing attention for demand-side factors by stimulating employers to employ workers from vulnerable groups. The involvement of employers is critical to the success of active labor market policies [9]. An example is job carving to adjust business processes; this is helpful for a successful and sustainable employment of vulnerable workers [10, 11]. However, relatively little is known about what factors influence organizations to hire vulnerable workers, more specifically the relation between motives, actually hiring and retention, and what is needed to influence this. Partly because many studies used the intention to hire people from vulnerable groups as a proxy for actual hiring, while validation of this conjecture is lacking (e.g., [12–14]).

Next to economic factors and job fit, other factors like social legitimacy/expectations and a good public image play an important role in hiring people from vulnerable groups [15]. Several studies have also focused on employers’ perception of the employability of vulnerable workers, but these produced conflicting results. Most employers express positive attitudes towards hiring vulnerable workers, but are often reluctant to actually hire them [16–18]. This is partly due to negative beliefs including concerns about work performance, safety, productivity, lack of employability and skills [18], and potential barriers such as a lack of financial support within the company [19, 20]. Another complicating factor in research is that differences in governmental support policies between and even within countries might affect employer perceptions of the employability of vulnerable groups.

Although there is some knowledge about motives to hire vulnerable workers and organizational factors that relate to hiring these workers, little is known how to convert hiring intentions of employers to actual employing vulnerable workers. An integrative framework to systematically examine inclusive employer behavior may be helpful to structure factors and relations, and thereby identifying targets for interventions. The Integrative Model of Behavioral Prediction provides such a framework [21]. This model is the update of the Theory of Reasoned Action and the Theory of Planned Behavior [22, 23], both of which focus on predicting the intention of people to behave in a certain way. The Integrative Model additionally includes the translation of intention into actual behavior. The Integrative Model could explain the conflicting results of previous studies and provide a better overview of the factors that affect employers’ hiring and retention of vulnerable workers. For instance, Fraser et al. used the Theory of Planned Behavior to predict intentions of employers (*N* = 92) to hire people with a disability [13]. They showed that employers had a much stronger intention to hire workers with a disability when employers (a) had a positive attitude toward workers with a disability, (b) thought that other employers deemed it important to hire workers with a disability (i.e., subjective norm), and (c) thought they were able to hire workers with a disability (i.e., behavioral control or self-efficacy). Araten-Bergman also showed that the attitude, subjective norm, and self-efficacy of employers (*N* = 250) predicted the intention of managers to hire people with a disability, but not the actual hiring behaviors [24]. The Integrative Model does include the translation of intention into actual behavior and describes that lack of skills to perform the hiring behaviors and contextual constraints that hinder this translation. Therefore, insights in skills and contextual constraints are also needed to determine which factors drive employers’ hiring behavior, aside from the intention, which is influenced by attitude, subjective norm and self-efficacy.

Our aim is to further explore the factors of the Integrative Model of Behavioral Prediction, that is, attitude, intention, skills and barriers, in relation to actual employment of vulnerable workers. This offers more insight into key demand-side factors associated with employing vulnerable workers, which may provide new leads for vocational rehabilitation interventions. We also examine differences in the relationships between factors of the Integrative Model and the hiring of vulnerable workers by size of organization. This is important since organizational factors, such as having an human resources-department in charge of recruiting, hiring and training, may result in different skills and contextual constraints between small and large organizations [25].

## 2 Method

### 2.1 Population

Data were obtained from the 2014, 2016 and 2019 Netherlands Employers Work Survey, a two-yearly survey that targets a representative sample of Dutch profit and non-profit organizations [26]. Organizations were randomly selected from the Netherlands National Job Information System. The selected organizations received a letter to participate with a unique code to fill out the questionnaire. A week after sending the questionnaire, organizations were contacted by telephone and requested to participate. During the phone call, it was verified whether the organization met the inclusion criterion (i.e., having at least one employee). The survey is normally filled in by managing directors (in small or medium organizations) or human resources managers (medium to large organizations). In total, 71,547 organizations were approached, of which 14,341 participated (response rate: 20.0%). The main reason for not participating was a lack of time or not wanting to participate, followed by finding the research uninteresting and the refusal of receptionists to transfer the call to an appropriate representative. We excluded organizations with missing values on the outcome variable (i.e. employment of vulnerable workers) (*N* = 948), and organizations with missing values on any of the other variables (except for satisfaction with employee flexibility, which was not measured in 2019) (*N* = 1,492). Furthermore, this study focused on regular employers, excluding sheltered work places (*N* = 64). This led to a study population of 11,837 organizations.

### 2.2 Employment of vulnerable workers

The behavioral outcome variable is whether or not an organization actually employed vulnerable workers. Organizations were dichotomized into an inclusive organization if at least 1% of the employees was from a vulnerable group, and other organizations were categorized as non-inclusive. This was based on the question: “Approximately how many employees in your branch of the organization are from a vulnerable labor market position?” People in this category were defined as young or partially-disabled people, the long-term unemployed or school dropouts.

### 2.3 Employer factors

Factors were either formulated in more general terms or explicitly related to vulnerable workers. The potential relevant factors in the Netherlands Employers Work Survey dataset were selected based on a literature review of factors associated with intention or actual hiring of vulnerable workers [27]. Factors measured with one item concerned categorical items and factors consisting of multiple items usually were 5-point Likert scales with good reliabilities. All factors were dichotomized. Table 1 provides a more detailed description.

**Table 1:** Description of the measurements of the employer factors

Measurement	Data transformation	Chronbach’s alpha
**Attitude**
Expected negative impact (none)	Expected negative impact due to employment of vulnerable groups: 1) cost of supervision/training; 2) financial risks due to productivity loss; 3) expected organizational adjustments	No negative impact vs negative impact on at least one item	NA
**Intention**
Having a mission	Employing people from vulnerable labor market groups is part of the mission statement	Yes vs no	NA
**Skills**
**General human resources skills**
Employer-employee negotiations	Employees can negotiate about 1) work and breaks; 2) working conditions and absenteeism	At least one (totally) agree vs none	α= 0.75
Personalization	It is possible to personalize 1) work performance; 2) development/training; 3) task content and/or number of tasks	At least one a (very) high degree vs none;	α= 0.73
Employee autonomy	Employees can decide 1) the work method; 2) the division of labor; 3) the working hours and breaks	At least one (totally) agree vs none	α= 0.79
Human resources practices
Employability	Employees are entitled 1) to invest personal employability budget; 2) to have regular performance and/or assessment interviews; 3) to promotions and career opportunities; 4) to education and training	At least one scheme vs none	NA
Working conditions	Employees are entitled 1) to commuting arrangements; 2) to human resources practices that lighten an employee’s financial burden; 3) for human resources practices allowing time for informal care; 4) to negotiate personal terms of employment	At least one scheme vs none	NA
Organizing working hours	Employees are entitled 1) to work part-time; 2) to flexible working hours; 3) to work from home	At least one scheme vs none	NA
Health &vitality	Human resources practices to maintain health and vitality are available	Yes vs no	NA
***Inclusive-related human resources skills***
Hiring problems regarding vulnerable groups	1) Unfamiliar with finding and recruiting people in vulnerable labor market groups; 2) people in vulnerable labor market groups do not apply at our organization	No barriers vs at least one barrier	NA
Inclusive human resources practices	Taking (or intending to take) the following actions: 1) creating workplaces for new contracts/tenders; 2) creating new workplaces from existing jobs; 3) providing work experience places, internships, and project-based learning; 4) hiring people in vulnerable labor market groups on a temporary basis (through agencies); 5) retrieving work lost to outsourcing and/or offshoring; 6) exploring with other employers in the region how to make work and tasks suitable for people with (employment) challenges or disabilities	At least one human resources practice vs none	NA
Human resources compensation options	1) financial compensation (premium deduction?) when hiring people over 50 years of age; 2) wage compensation in case of illness of hired unemployed people over 50; 3) no-risk policy in the event of illness and incapacity (for work); 4) grants for adjusting the workplace; 5) external coach (compensation for additional counseling at work); 6) trial placement for up to 3 months without having to pay wages; 7) wage dispensation for people that were disabled before the age of 30; 8) financial compensation or exemptions when employing people with a disability; 9) municipal wage grants.	Having used at least one Dutch compensation scheme vs none	NA
**Barriers**
***Economic factors***
Financial results	The last two years. 1) productivity has been above average; 2) turnover has been above average; 3) profits/positive financial results have been above average	At least one above average vs none	α= 0.80
Economic identity	Organization has a profit or a non-profit character	Non-profit vs profit or combination	NA
Quality of production	The last two years 1) the quality of our products/services has been above average; 2) customer satisfaction has been above average	At least one above average vs none	α= 0.75
Employment opportunity	Current number of employees compared to two years ago	Increased at least 5% vs stable or decreased	NA
Outstanding vacancies	Outstanding vacancies	Yes vs no or don’t know	NA
***Type of work***
Satisfaction with flexibility of employees	1) Satisfaction with employability; 2) flexibility in working hours; 3) willingness to learn new things	At least one to a (very) high degree vs none	α= 0.78
Repetitive work	Repetitive work as occupational risk factor	Yes vs no	NA
Physically demanding work	Physically demanding work (lift, push and/or pull) as occupational risk factor	Yes vs no	NA
Emotionally demanding work	Emotionally demanding work as occupational risk factor	Yes vs no	NA
Operational work	Proportion of operational work	At least 70% operational staff vs less than 70%	NA
Work tasks suitable for people with (employment) challenges or a disability	Work tasks in the organization are not appropriate for people with (employment) challenges or a disability	No vs yes	NA

#### 2.3.1 Attitude

The attitude towards employing vulnerable workers was measured by three questions on expected negative impact (cost of supervision/training, financial risks due to productivity loss, and organizational adjustments), and dichotomized into no impact and at least one negative impact.

#### 2.3.2 Intention

The intention to employ vulnerable workers was measured by the question: is employing vulnerable groups explicitly part of the organization’s mission statement (yes/no)?

#### 2.3.3 Skills

Skills of the organization were mainly interpreted as factors that affected employees and, as such, the actual hiring vulnerable workers. They were measured on both a general level and more specifically related to vulnerable workers. The general human resources skills of the organization focused on four general human resources policies: (1) negotiation between employer –employee (2 items), (2) personalization (3 items), (3) employee autonomy (3 items), and (4) human resources practices concerning employability (4 items), working conditions (4 items), organizing working hours (1 item) and health and vitality (1 item).

The skills specifically related to vulnerable workers focused on three factors: (1) Hiring problems regarding vulnerable groups, (2) inclusive-related human resources practices and (3) human resources compensation options. *Hiring problems* were measured with two questions on familiarity with finding and recruiting people in vulnerable labor market groups and by the belief that people in a vulnerable labor market group do not apply at their organization (no problem vs at least one problem). Human resources *compensation options* was measured using six items (e.g., no-risk policy in the event of illness and incapacity for work) and *Inclusive* human resources *practices* (e.g., creating new workplaces from existing jobs) using nine items. The factors were dichotomized as no human resources compensation option or inclusive human resources practice and at least one option or practice, respectively.

#### 2.3.4 Barriers

Barriers were construed as factors that could turn out to be an internal organizational barrier for actually hiring vulnerable workers. We discerned two potential barriers: Economic factors and Type of work. *Economic factors* entailed five factors: (1) financial results (3 items), (2) economic identity (1 item), (3) quality of production (2 items), (4) employment opportunity (1 item), and (5) outstanding vacancies (1 item). *Type of work* included six factors: (1) satisfaction with flexibility of employees (3 items), (2) repetitive work (1 item), (3) physically demanding work (1 item), (4) emotionally demanding work (1 item), (5) operational work (1 item), and (6) work tasks suitable for people with (employment) challenges or a disability (1 item).

### 2.4 Data analyses

Logistic regression models were used to examine the relationship between employer factors and employment of vulnerable workers (less than 1% of employees in the organization was from a vulnerable group vs at least 1% of the employees in the organization was from a vulnerable group). Analyses were adjusted for known organizational characteristics that affect the employment of vulnerable groups (Hazelzet et al., 2017): a) number of employees, (b) educational level in percentage of employees with low (intermediate secondary education or less), intermediate (intermediate vocational or higher secondary education), and high (higher vocational education or university) educational level, (c) age of employees into < 25 years, 25–44 years, 45–54 years, and≥55 years, and (d) sector into twelve categories based on the Netherlands Standard Industrial Classifications 2008. We also adjusted for the year that organizations filled in the questionnaire.

The impact of size of organization was assessed using interaction terms between size of organization and each employer factor, and by stratifying the analyses by size of organization (< 25 employees, 25–99 employees,≥100 employees). Bonferroni adjustments were applied to all analyses in order to reduce the risk of Type I errors, such that the significance criteria were *P* < 0.0022 for all analyses. The analyses were performed using SPSS version 25.

## 3 Results

Organizations had a median number of 28 employees (IQR: 9–100), and on average a similar percentage of employees with low and high educational levels. Of the 11,837 organizations, 5,601 (47%) were inclusive organizations and 6,236 (53%) organizations were not inclusive. Inclusive organizations were larger in size, had employed slightly more low-educated people, and the mean age of employees was similar in comparison with non-inclusive organizations (Table 2).

**Table 2:** General characteristics of the organizations

	Inclusive organizations *n* = 5,601	Non-inclusive organizations *N* = 6,236
**Size establishment of the organization (%, (n))**
Number of employees (median, IQR)	60 (20–126)	14 (6–53)
1–9 employees	12% (672)	41% (2,523)
10–49 employees	32% (1,815)	33% (2,051)
50–99 employees	21% (1,174)	10% (596)
100 + employees	35% (1,940)	17% (1,066)
**Educational level of employees (%, (n))**
Percentage low educated	32% (30)	25 (32)
Percentage intermediate educated	40% (27)	43 (33)
Percentage high educated	28% (30)	32 (35)
**Age of employees (% (n))**
Percentage<25 years	15% (19)	14% (20)
Percentage 25–44 years	40% (20)	43% (25)
Percentage 45–54 years	29% (17)	28% (22)
Percentage≥55 years	16% (14)	15% (18)
**Sector (%, (n))**
Agriculture	2% (90)	2% (111)
Manufacturing	20% (1,102)	16% (997)
Construction	8% (422)	6% (355)
Wholesale and retail trade	12% (691)	15% (916)
Hospitality industry	6% (312)	4% (221)
Transportation and storage	7% (381)	8% (513)
Financial institutions	3% (142)	5% (321)
Service industry	14% (789)	18% (1,124)
Public services	3% (165)	2% (149)
Education	10% (547)	9% (548)
Health and wellbeing	12% (651)	10% (621)
Other service activities	6% (309)	6% (360)

Abbreviations: IQR: interquartile range; SD: standard deviation.

### 3.1 Attitude

Of the inclusive organizations 61% expected no negative impact related to cost of supervision, productivity loss or necessary organizational adjustments of employing vulnerable groups, whereas 70% of the non-inclusive organizations expected no negative impact (Table 3). In multivariate-adjusted analyses more expected negative impact was positively associated with employment of vulnerable workers (OR: 0.81).

**Table 3:** Relationships between attitude, intention, skills and barriers, and employment of vulnerable workers

	Unadjusted percentage (number)		Odds ratios
		(95% confidence
		intervals)
	Inclusive organizations *n* = 5,601	Non-inclusive organizations *N* = 6,236
**Attitude**
Expected negative impact (none)	61% (3,412)	70% (4,336)	**0.81 (0.74–0.88)**
**Intention**
Having a mission (yes)	40% (2,104)	8% (453)	**6.09 (5.42–6.84)**
**Skills**
***General human resources skills***
Employer-employee negotiations (yes)	90% (5,040)	84% (5,222)	**1.49 (1.32–1.68)**
Personalization (yes)	85% (4,769)	80% (4,958)	**1.52 (1.37–1.69)**
Employee autonomy (yes)	57% (3,178)	62% (3,878)	1.10 (1.02–1.20)
Human resources practices
Employability (yes)	98% (5,505)	95% (5,915)	**1.78 (1.39–2.29)**
Working conditions (yes)	76% (4,266)	64% (4,013)	**1.40 (1.27–1.53)**
Organizing working hours (yes)	95% (5,317)	90% (5,589)	**1.66 (1.41–1.95)**
Health &vitality (yes)	35% (1,941)	25% (1,571)	1.08 (0.99–1.18)
***Inclusive-related human resources skills***
Hiring problems regarding vulnerable groups (none)	83% (4,649)	74% (4,612)	**1.87 (1.70–2.06)**
Inclusive human resources practices (yes)	67% (3,743)	24% (1,484)	**4.83 (4.43–5.27)**
Human resources compensation options (yes)	67% (3,773)	26% (1,621)	**4.45 (4.09–4.85)**
**Barriers**
***Economic factors***
Financial results (good)	68% (3,823)	60% (3,760)	**1.32 (1.21–1.43)**
Economic identity (non-profit)	26% (1,474)	19% (1,198)	**1.35 (1.17–1.55)**
Quality of production (good)	64% (3,571)	61% (3,809)	**1.16 (1.07–1.26)**
Employment opportunity (increased)	19% (1,087)	20% (1,259)	**1.33 (1.22–1.45)**
Outstanding vacancies (yes)	55% (3,068)	35% (2,220)	**1.37 (1.25–1.49)**
***Type of work***
Satisfaction with flexibility employees (satisfied) ^1^	83% (6,858)	88% (3,704)	0.91 (0.80–1.05)
Repetitive work (risk factor)	12% (685)	7% (436)	**1.31 (1.14–1.51)**
Physically demanding work (risk factor)	47% (2,631)	34% (2,147)	**1.19 (1.09–1.30)**
Emotional demanding work (risk factor)	66% (3,714)	59% (3,691)	1.18 (1.03–1.34)
Operational work (≥70% operational work)	65% (3,610)	55% (3,425)	**1.15 (1.06–1.25)**
Work tasks suitable for people with (employment) challenges or a disability	50% (2,799)	42% (2,607)	**1.40 (1.27–1.54)**

Odds ratio’s adjusted for size organization, percentage low educated employees, percentage employees younger than 45 years, sector, and year. Boldface indicates statistical significance at *P* < 0.0022. ^1^Not measured in 2019 (*n* = 7,998).

### 3.2 Intention

We observed that 40% of the inclusive and 8% of the non-inclusive organizations had social inclusion integrated in their mission statement. The analysis showed that the odds of having a mission statement regarding social inclusion were six times higher in inclusive organizations than in non-inclusive organizations (OR: 6.09).

### 3.3 Skills

The analyses showed that five of the seven factors regarding general human resources skills were related to employment of vulnerable workers (Table 3). Inclusive organizations more often offered the possibility of negotiating working hours and absenteeism (OR: 1.49), the possibility to personalize work performance, development, and job content (OR: 1.52), and the presence of human resources practices regarding employability (OR: 1.78), working conditions (OR: 1.40) and organizing working hours (OR: 1.66) in comparison to non-inclusive organizations.

All three inclusive-related human resources skills were associated with employment of vulnerable workers. Inclusive organizations more often experience no hiring problems (OR: 1.87), often had more inclusive human resources practices (OR: 4.83), and often initiated more supportive human resources actions (OR: 4.45).

### 3.4 Barriers

Inclusive organizations experienced less economic constraints in employing vulnerable workers than non-inclusive organizations. They had better financial results in the last two years (OR: 1.32), better quality of production (OR:1.16), more often an increase in number of employees over the last two years (OR: 1.33), and more outstanding vacancies (OR: 1.37).

With regard to type of work, we observed that inclusive organizations more often had a lot of operational work (OR: 1.15), and repetitive (OR: 1.31) and physically demanding work (OR: 1.19) compared to non-inclusive organizations. There was no difference in satisfaction with flexibility of employees and amount of emotionally demanding work between organizations.

### 3.5 Differences by size of organization

The strength of the relationship between some of the employer factors and employment vulnerable workers differed by the size of the organization (*P*-value for interaction < 0.0001) (Table 4). The relationships between human resources practices regarding employability, economic identity, and having outstanding vacancies with employment of vulnerable workers was stronger and only statistically significant in small organizations compared to medium and large organizations. Also, having a mission statement regarding social inclusion, inclusive human resources practices and using human resources compensation options were in small organizations more strongly related to employment of vulnerable workers than in medium or large organizations. Having work tasks that are suitable for vulnerable workers was only related to employment of these groups in small and medium organizations and not in large organizations.

**Table 4:** Relationships between employer factors and inclusive organizational behavior stratified by size of organization

	Size organization < 25 employees (47%)	Size organization < 25–99 employees (28%)	Size organization≥100 (25%)	*P*-value for interaction between determinant and size of organization
**Attitude**
Expected negative impact (none)	**0.76 (0.67–0.86)**	**0.70 (0.60–0.81)**	0.90 (0.77–1.05)	0.041
**Intention**
Having a mission (yes)	**15.58 (12.42–19.55)**	**6.82 (5.40–8.61)**	**2.71 (2.28–3.23)**	< 0.0001
**Skills**
***General human resources skills***
Employer-employee negotiations (yes)	**1.48 (1.24–1.75)**	**1.43 (1.14–1.80)**	1.45 (1.11–1.90)	0.998
Personalization (yes)	**1.67 (1.42–1.96)**	1.27 (1.04–1.55)	**1.51 (1.20–1.88)**	0.525
Employee autonomy (yes)	1.20 (1.06–1.37)	1.04 (0.90–1.22)	1.12 (0.95–1.32)	0.210
Human resources practices
Employability (yes)	**1.72 (1.30–2.29)**	1.08 (0.53–2.18)	2.64 (0.82–8.48)	< 0.0001
Working conditions (yes)	**1.45 (1.28–1.65)**	1.28 (1.07–1.53)	1.17 (0.93–1.48)	< 0.0001
Organizing working hours (yes)	**1.59 (1.06–2.36)**	1.30 (0.91–1.86)	1.93 (1.12–3.32)	0.085
Health &vitality (yes)	1.22 (1.04–1.45)	1.15 (0.98–1.34)	0.86 (0.73–1.00)	< 0.0001
***Inclusive-related human resources skills***
Hiring problems regarding vulnerable groups (none)	**1.74 (1.49–2.03)**	1.89 (1.59–2.25)	1.79 (1.50–2.14)	0.149
Inclusive human resources practices (yes)	**6.38 (5.57–7.30)**	4.57 (3.91–5.36)	3.52 (2.98–4.17)	< 0.0001
Human resources compensation options (yes)	**5.54 (4.93–6.43)**	3.90 (3.35–4.55)	3.81 (3.22–4.51)	< 0.0001
**Barriers**
***Economic factors***
Financial results (good)	**1.34 (1.18–1.52)**	**1.30 (1.12–1.52)**	1.20 (1.01–1.41)	0.922
Economic identity (non-profit)	**1.46 (1.16–1.85)**	1.31 (1.02–1.68)	1.20 (0.91–1.57)	< 0.0001
Quality of production (good)	**1.24 (1.09–1.41)**	1.10 (0.95–1.28)	1.12 (0.96–1.31)	0.778
Employment opportunity (increased)	**1.49 (1.30–1.71)**	1.12 (0.95–1.32)	**1.33 (1.12–1.58)**	0.624
Outstanding vacancies (yes)	**1.60 (1.39–1.84)**	1.22 (1.05–1.43)	1.13 (0.94–1.36)	< 0.0001
***Type of work***
Satisfaction with flexibility employees (satisfied) ^1^	0.75 (0.59–0.94)	0.96 (0.75–1.23)	1.04 (0.81–1.34)	0.043
Repetitive work (risk factor)	1.41 (1.11–1.79)	1.20 (0.94–1.53)	1.18 (0.92–1.51)	0.199
Physically demanding work (risk factor)	1.16 (1.01–1.33)	1.16 (0.98–1.38)	1.12 (0.94–1.34)	0.156
Emotional demanding work (risk factor)	1.34 (1.07–1.68)	1.10 (0.88–1.39)	1.14 (0.90–1.43)	< 0.0001
Operational work (≥70% operational work)	1.08 (0.95–1.22)	1.12 (0.96–1.31)	1.23 (1.04–1.44)	0.162
Work tasks suitable for people with (employment) challenges or a disability	**1.58 (1.40–1.78)**	**1.60 (1.38–1.85)**	1.02 (0.87–1.19)	< 0.0001

Model adjusted for percentage low educated employees, percentage employees younger than 45 years, sector, and year. Boldface indicates statistical significance at *P* < 0.0022. ^1^Not measured in 2019 (*n* = 7,998).

## 4 Discussion

The results of the present study indicate that the Integrative Model of Fishbein and Ajzen is useful to understand the behavior of organizations. The concepts of this model distinguished well between organizations that employ vulnerable workers and those who do not. Earlier findings showed that attitude, subjective norms and self-efficacy are related to employers’ intentions to hire people from vulnerable labor market groups [13, 24]; our results expand on that by showing that attitude, intention, skills and barriers are related to actually employing vulnerable groups. A stronger intention, more skills and less barriers were positively related to employing vulnerable workers. The results differed for the size of the organization. Intention, several barriers and skills were more important for employing vulnerable workers in small organizations than in large ones.

In contrast to previous studies which found that a positive attitude was related to a stronger intention to hire people from vulnerable groups [18–20], we observed that inclusive organizations expected more negative impact than non-inclusive organizations. One explanation is our operationalization of attitude. We measured negative financial expectations and a set of items restricted to costly supervision, productivity loss and organizational adjustments. In contrast, studies that found a positive attitude to be related to hiring people with a disability measured other aspects of attitude, such as positive expectations (e.g., better social responsible image), a more comprehensive set of negative expectations (e.g., concerns about co-workers’ reactions to employees from vulnerable groups) [20], and a lack of employability and skills [18]. Inclusive organizations may also intrinsically value employment of vulnerable groups, despite expected negative impacts. Another explanation is that we investigated actual employing behavior and previous studies mostly intention to hire. The inclusive organizations in the present study may have more realistic insights into the negative impact of employing people from vulnerable groups which would explain their more negative attitude compared to non-inclusive organizations. Although inclusive organizations have a more negative attitude, they still employed and also intended to hire vulnerable workers. This stronger intention is probably explained by other factors that influence their intention, such as self-efficacy and subjective norms.

According to the Integrative Model of Fishbein and Ajzen, a person’s intention to perform a behavior can be converted into actual behavior if one has the skills and can overcome contextual constraints [21]. Our results showed that inclusive-related human resources skills have a strong impact on actually employing vulnerable workers. Organizations that are less familiar with governmental incentives, supporting services and how people from a vulnerable labor market groups can be recruited, and those organizations that are less able to adapt working conditions to those people were less likely to employ vulnerable workers. Previous studies also indicate that employment of those people is hampered by a lack of awareness of the use of legal issues and governmental support to promote and facilitate employment of vulnerable workers [10, 28–30]. Aside from inclusive-related human resources skills, our results imply that some general human resources policies and practices are also beneficial in employing people from vulnerable groups. It seems that inclusive organizations have more adequate facilities to employ vulnerable workers and are better in overcoming hurdles, such as personalizing work performance, development and task content. With regard to barriers, we observed that inclusive organizations have better economic conditions (e.g., financial results and employment opportunity) than non-inclusive organizations. Therefore, well-performing organizations have more possibilities and are possibly more willing to employ vulnerable workers. Furthermore, human resources skills as well as economic constraints seem to be particularly important for small to medium organizations. Fraser et al.also found that small organizations had a lower self-efficacy than larger organizations [25]; they believed that senior management was less committed to hire workers from vulnerable groups, had fewer job openings and were less likely to receive tax credits for hiring these workers compared to large organizations.

Currently, there is little focus of vocational rehabilitation on demand-side factors, but the Integrative Model of Fishbein and Ajzen supports a systematic analysis of the factors that influence employing vulnerable workers, which offers opportunities to better target interventions. For rehabilitation professionals our results imply that in particular small and medium organizations may benefit from their help and support. Organizations willing to hire vulnerable workers but lacking skills, experience or perceiving barriers can be supported by rehabilitation professionals by, for example, making them aware of economic incentives and helping them with the recruitment process. The review of Bonaccio et al. also describes that recruiting qualified people with a disability is a challenge for organizations [31], and that community-based organizations specialized in supporting employment needs of people in vulnerable groups play a key role in a successful hiring process. For example, they can assist in job advertising and ensuring that recruitment processes and messages do not act as barriers [28, 31] highlight that potentially effective hiring and retention practices, such as using available tax incentives, internship opportunities, mentor programs, and formation of disability-focused employee resource groups or networks are hardly used and could be introduced by vocational rehabilitation agencies. For employers that do not intend to hire people from vulnerable labor market groups, rehabilitation professionals could increase the intention by influencing the attitude toward hiring people from vulnerable labor market groups, subjective norm and self-efficacy [13, 24]. Insight into the motivations, preferences and needs of organizations are also required to successfully influence the intention of employers [9].

The strength of the present study is the use of a large dataset of employers across all Dutch sectors, making it possible to analyze differences by size of organizations. This is one of the first explorative studies into the use of the Integrative Model of Fishbein and Ajzen to structure employer factors and employment behavior [21]. It shows that the Integrative Model is useful in explaining employment of vulnerable workers. Future research is needed to validate other aspects of the model, like subjective norm, self-efficacy, and other barriers (e.g., organizational culture) and skills. The relatively low response rate (20%) is a limitation of the study. As organizations of all sectors were still included and we also investigated differences by size of organization, our results are likely to be representative for Dutch organizations. A third limitation is that the perceptions of a single person within an organization filling in the questionnaire about employer factors may be limited, and the self-reported measures may be susceptible to socially desirable answers, especially as it relates to a vulnerable population. This may have led to an underestimation of the observed relationships. Employer factors may differ upon specific groups. Yet, in our study we could not distinguish between specific vulnerable groups. Fourth, as we compared inclusive organizations with non-inclusive organizations, we could not assess how employer factors differ between different kind of inclusive organization. For example, between organizations with many or few vulnerable employees, or between organizations that employ people from different vulnerable groups. It seems likely that the more vulnerable workers an organization has the larger the differences become with non-inclusive organizations. Further research is needed to examine whether our results differ according to the type of disability (e.g. physical compared to mental disabilities). Finally, due to the cross-sectional nature of our study, causal inferences cannot be made. This is especially the case for inclusive-related human resources skills, of which we do not know whether better skills facilitate hiring vulnerable workers or whether organizations improve their skills because they hire those people. Longitudinal research is needed to clarify the temporal relationship.

## 5 Conclusion

We found that all included concepts of the Integrative Model of Fishbein and Ajzen were associated with employment of vulnerable workers. This supports the use of the Integrative Model to understand employers’ behavior and to provide an overview of possible targets for interventions on demand-side factors in vocational rehabilitation. We observed several demand-side factors related to the employment of vulnerable workers that were particularly important for small to medium organizations. The findings indicate that vocational rehabilitation interventions need to pay particular attention to employers’ barriers regarding the economic condition of the organization and skills to recruit vulnerable workers, the use of government incentives and supporting services, and to personalize and adapt jobs to fit the needs of vulnerable workers. Future research is needed to determine which demand-side strategies for vocational rehabilitation professionals are effective to increase the intention of employers by influencing attitude, subjective norms and self-efficacy, and which strategies are effective to improve skills and lower barriers of employers who have the intention of hiring vulnerable workers.
